# Exploring the Relationship Between Adipocytokines and Endometrial Cancer: Identifying Correlations With Clinico‐Pathological Prognostic Factors

**DOI:** 10.1002/cam4.71007

**Published:** 2025-07-11

**Authors:** Irene Ray, Carla S. Möller‐Levet, Agnieszka Michael, Simon Butler‐Manuel, Jayanta Chatterjee, Anil Tailor, Ben Haagsma, Izhar Bagwan, Lisiane B. Meira, Patricia E. Ellis

**Affiliations:** ^1^ Department of Clinical and Experimental Medicine University of Surrey Guildford UK; ^2^ Academic Department of Gynaecological Oncology Royal Surrey NHS Foundation Trust Guildford UK; ^3^ Bioinformatics Core Facility University of Surrey Guildford UK; ^4^ Department of Oncology Royal Surrey NHS Foundation Trust Guildford UK; ^5^ Department of Life Sciences Brunel University of London Uxbridge UK; ^6^ Department of Histopathology Royal Surrey NHS Foundation Trust Guildford UK

**Keywords:** adipocytokines, endometrial cancer, immunohistochemistry, qPCR

## Abstract

**Background:**

Endometrial cancer, a malignancy linked with obesity, may be influenced by adipocytokines signalling due to chronic inflammation. This study explores the molecular expression patterns of adiponectin, leptin, interleukin6 (IL6), tumor necrosis factor (TNF)α, and their receptors in endometrial cancer patients and associations with lymphovascular space invasion (LVSI) and other tumour characteristics.

**Methods:**

We analysed mRNA expression levels of the above biomarkers in endometrial cancer tissue using quantitative reverse transcriptase polymerase chain reaction (qRT‐PCR), comparing them to benign endometrial tissue controls. Additionally, expressions in adipose tissue and lymph nodes were assessed, with correlations drawn between biomarker expression, patient demographics, and tumor characteristics.

**Results:**

Using qRT‐PCR analysis, endometrial cancer tissues (*n* = 39) exhibited higher expression levels of adiponectin, leptin, IL6, TNFα, and their receptors, IL6R and TNFRSF1A/B, compared to the calibrator sample, which consisted of five pooled benign endometrial control samples. Intriguingly, the adiponectin receptors, ADIPOR1 and ADIPOR2 demonstrated opposing correlations with cancer characteristics such as grade, histology, LVSI, and microcystic elongated and fragmented (MELF) pattern. LVSI was linked to increased levels of markers such as IL6R and ADIPOR2, along with decreased expressions of OBR (leptin receptor) and ADIPOR1, suggesting their potential as surrogate markers for diagnosing LVSI. Notably, higher adiponectin expression was observed in the cancerous lymph nodes of patients with LVSI, contrasting with those without LVSI.

**Conclusion:**

This study provides novel insight into differential role of adiponectin receptors in endometrial cancer and the associations of various markers with LVSI, emphasizing the need for tissue‐specific biomarker assessments in determining treatment strategies.

## Graphical abstract

Endometrial cancer is linked to obesity and chronic inflammation, potentially mediated by adipocytokines like adiponectin, leptin, IL6, and TNFα. This study examined mRNA expression of these markers and their receptors in cancerous versus benign endometrial tissue using qRT‐PCR. Cancer tissues demonstrated differential expression of these markers compared to benign controls. Notably, ADIPOR1 and ADIPOR2 showed opposite correlations with cancer traits like grade, histology, and LVSI. LVSI was associated with increased IL6R and ADIPOR2, and reduced OBR and ADIPOR1, suggesting their potential as additional diagnostic markers for LVSI. Notably, higher adiponectin expression was observed in the cancerous lymph nodes of patients with LVSI, contrasting with those without LVSI.

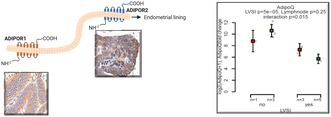

**Abbreviations**
AMPK: AMP‐activated protein kinase
BMI: body mass index
IHC: immunohistochemistry
IL6: interleukin6
JAK/STAT3: janus kinase/activator of transcription 3
LVSI: lymphovascular space invasion
MEK–ERK: MAPK–ERK kinase/extracellular signal‐regulated kinase
MELF: microcystic elongated and fragmented
MSI: microsatellite instability
PI3K/Akt: phosphoinositide 3‐kinase
qRT‐PCR: quantitative reverse transcriptase polymerase chain reaction
Ras–Raf: rat sarcoma protein/rapidly accelerated fibrosarcoma protein
TCGA: the cancer genome atlas
TNFα: tumor necrosis factor α

## 1 Introduction

Endometrial cancer comprises 96% of uterine cancers, with a 59% increase in incidence among UK women since the early 1990s [1]. Mortality rates are projected to rise by 12% in the UK between 2023–2025 and 2038–2040 [1]. Despite a 5‐year survival rate of about 80%, significant prognostic differences exist across histological types, necessitating precise risk stratification [2].

Traditionally, risk stratification for endometrial cancer was based on clinico‐pathological features such as histopathologic type, grade, and stage, including depth of myometrial invasion and lymph nodal involvement [3]. Historically, endometrial cancers have been classified histologically into two subtypes: Type 1 and Type 2. Type 1 endometrial cancers are most common, typically hormone sensitive, of lower stage and grade, and have an excellent prognosis. In contrast, type 2 cancers are typically higher grade, with a higher recurrence rate even at early stages. Surgery is the cornerstone of treatment, crucial for staging and guiding further adjuvant treatments for high‐risk patients. However, risk stratification using clinical factors evaluated after surgery cannot inform pre‐surgical decision‐making [3].

Incorporating molecular subtypes alongside histological classification enhanced understanding of cancer origins, prognosis, and treatment responses, especially for high‐risk endometrial cancer cases [4]. Recent molecular subtyping by the Cancer Genome Atlas (TCGA) group has identified various molecular sub‐types of endometrial cancer, which has led to a new classification, developed by the Leiden/TransPORTEC group [5, 6]. This classification divides endometrial cancer into four subgroups, *TP53* mutated, microsatellite unstable, *POLE* mutated and a fourth group that demonstrated none of these molecular profiles and hence classified as ‘no specific molecular profile’, which is actually the most common sub‐type. Although this molecular classification has an advanced understanding of genetics and pathophysiology of endometrial cancer sub‐types, it remains limited in addressing indeterminate subtypes, labelled as ‘no specific molecular profile’ [7]. This highlights a still unknown gap in molecular subtyping that could be bridged by linking other biomarkers and molecular pathways [8, 9].

Obesity forms a part of the spectrum of the metabolic syndrome that is closely related to endometrial cancer, and hence investigating molecular pathways affected by obesity could help in identifying markers that could have prognostic potential. Obesity increases endometrial cancer risk via chronic inflammation and altered adipocytokine levels with mitogenic potential, promoting cancer cell proliferation, apoptosis inhibition, and neo‐angiogenesis [10]. Adipocytokines encompass adipokines and cytokines. Adiponectin is the most abundant adipokine and has been implicated in the pathogenesis of insulin resistance and diabetes [11]. Acting via its receptors, ADIPOR1 and ADIPOR2, and the AMP‐activated protein kinase (AMPK) pathway, adiponectin has been reported to be involved in lipid and glucose metabolism and also to have anti‐angiogenic, anti‐inflammatory, and anti‐apoptotic properties [12, 13]. Leptin, another well‐known adipokine, is primarily associated with food intake and energy expenditure [11] and acts via its receptor, ObR, which works by activating Janus Kinase/Activator of Transcription 3 (JAK/STAT3), Rat Sarcoma protein/Rapidly Accelerated Fibrosarcoma protein/MAPK–ERK Kinase/Extracellular Signal‐Regulated Kinase (Ras–Raf–MEK–ERK) and the phosphoinositide 3‐kinase (PI3K/Akt) signalling pathways [14]. Leptin has contrasting biological functions to adiponectin—it has been shown to promote cell proliferation, angiogenesis, and metastasis [15]. In fact, a lower adiponectin/leptin ratio, or a higher leptin/adiponectin ratio, has been linked to an increased risk of endometrial cancer [16, 17]. Furthermore, among the cytokines, a shift of balance between pro‐ and anti‐inflammatory cytokines in the tumor microenvironment has been implicated in the pathogenesis of endometrial cancer. The cytokine IL‐6 has been reported to act mainly through its receptor IL6R and the JAK/STAT3, Ras/Raf/MEK/ERK, and the PI3K/Akt signalling pathways, which are involved in tumor proliferation, survival, and angiogenesis [18], whereas another prominent cytokine, TNFα, has been reported to activate the nuclear factor‐κB (NF‐κB) and c‐Jun NH2‐terminal kinase (JNK) signalling pathways acting via its designated receptors, TNFRSF1A and TNFRSF1B [19].

Pro‐inflammatory adipocytokines such as leptin and IL‐6 contribute not only to cancer development but also to endometrial cancer progression through multiple mechanisms. They promote cell proliferation via JAK/STAT3, MAPK/ERK, and PI3K/AKT pathways, and enhance migration and invasion by inducing epithelial‐mesenchymal transition (EMT), marked by loss of E‐cadherin and gain of N‐cadherin and vimentin [20, 21]. EMT, further promoted by Receptor Activator of Nuclear Factor‐κB/Ligand (RANK/RANKL) and Transforming Growth Factor‐β1 (TGF‐β1) increases cancer cell motility [22]. Additionally, leptin and IL‐6 upregulate matrix metalloproteinases (MMP‐2 and MMP‐9), facilitating extracellular matrix degradation and invasion [21, 23]. Additionally, they accelerate cell cycle progression by increasing cyclin D1 and decreasing p21, while suppressing apoptosis through downregulation of caspase‐3 and upregulation of Bcl‐2 and Mcl‐1 [24, 25]. These effects collectively drive tumor growth, metastasis, and resistance to cell death, contributing to disease progression and poor prognosis.

Molecular biomarkers play a crucial role in regulating cellular functions, and their dysregulation can increase cancer risk [26]. These biomarkers, generated by cells in response to diverse conditions, are detectable in bodily fluids like blood or urine, as well as in tissues [27]. In our previous publication, we linked high circulating levels of adiponectin with a 64.2% decreased risk of endometrial cancer (Odds Ratio = 0.358, 95% CI: 0.216–0.594, *p* = 0.0002) [28]. Intriguing associations were noted between circulating adiponectin levels and lymphovascular space invasion (LVSI) [28]. This study extends those findings by analyzing mRNA expressions of adiponectin, leptin, and their receptors in cancerous and benign endometrial tissues, adipose tissue, and lymph nodes. We also sought correlation between marker expressions and body mass index (BMI), patient demographics, and endometrial cancer characteristics, namely grade, stage, histology, LVSI, microcystic elongated fragmented pattern (MELF) and micro‐satellite instability (MSI). LVSI, that is, presence of tumor invasion in lymph and blood vessels beyond the tumor tissue, is an independent risk factor for cancer recurrence [29, 30]. MELF, a pattern of tumor invasion involving microcystic, elongated, and fragmented cells, is associated with adverse histological findings such as larger tumor size, deeper myometrial invasion, and LVSI [31]. MSI indicates a faulty DNA mismatch repair system that promotes cancer development and progression [32]. We measured the expressions of adiponectin, leptin, and their receptors in fresh parametrial adipose tissue and of all the markers and their receptors in some sentinel lymph nodes as well. By integrating adipocytokine profiling with histological and molecular features, this study aims to uncover biomarker‐driven patterns linked to tumor aggressiveness and improve pre‐surgical risk stratification. We followed the REMARK (Reporting Recommendations for Tumor Marker Prognostic Studies) reporting guidelines for biomarkers for presenting all information in the materials and methods, study design, statistical calculations, results, and discussion sections [33].

## 2 Materials and Methods

### 2.1 Ethics Approval and Participant Recruitment

Approval for the study was granted by the Health Research Authority and Health and Care Research Wales, UK and the patients were recruited from the gynaecological oncology and gynaecology departments of the Royal Surrey NHS Foundation Trust in Guildford, UK. Endometrial cancer patients (study group) underwent Da Vinci robotic‐assisted hysterectomy, bilateral salpingo‐oophorectomy, peritoneal washings, and sentinel node dissection, or dissection of pelvic and para‐aortic lymph nodes followed in some instances by chemotherapy and/or radiotherapy. The control patients included patients referred to the benign gynaecology department for benign reasons such as prolapse, fibroid uterus, or post‐menopausal bleeding, some of whom underwent either abdominal or vaginal hysterectomy. Further details of the two populations have been discussed in our previous publication [28].

### 2.2 Tissue Collection, Preparation and Storage

Fresh endometrial tumor tissue was collected from 39 patients with endometrial cancer in the study cohort and from 5 patients in the benign control cohort for comparison for qPCR analysis [28]. This tissue was obtained from the histology laboratory following an initial visual examination of the specimen by the histopathologist. The five benign control samples were pooled to create a calibrator sample and utilised as the calibrator sample for all the qPCRs.

Additionally, fresh adipose tissue samples were obtained from the parametrium of all patients with endometrial cancer, intra‐operatively.

These fresh endometrial and fat tissue specimens, approximately measuring 0.5 cm × 0.5 cm, were carefully preserved in aliquots containing 1 mL of RNAlater stabilisation solution (Life Technologies Limited, #AM7020, Waltham, MA, USA). After incubating at room temperature for an hour, they were stored frozen at −80°C.

Furthermore, formalin‐fixed paraffin‐embedded (FFPE) blocks of lymph nodes were collected from 12 patients within the study population who had undergone sentinel lymph node dissection (4 with cancer metastasis and 8 without cancer metastasis) for RNA extraction. Additionally, 10 FFPE blocks of endometrial cancer tissue and 10 benign endometrial tissues were obtained for immunohistochemical evaluation. The FFPE blocks were stored at room temperature. The 10 blocks were collected at random from the study and control populations, and only 10 were collected due to constraints of resources.

### 2.3 mRNA Extraction and Quantitative Reverse Transcriptase‐PCR (qPCR)

RNA was extracted from the fresh endometrial tissue using the PARIS kit (ThermoFischer Scientific, #AM1921, Waltham, MA, USA), from the adipose tissue using TRIzol Reagent (ThermoFischer Scientific, #15596018) and from the FFPE blocks of lymph nodes using the RecoverAll Total Nucleic Acid Isolation Kit (ThermoFischer Scientific, #AM1975). Isolated total RNA was subsequently treated with DNase using RQ1 RNase‐free DNase treatment kit (Promega, #M6101, Chilworth, Southampton, UK). The first strand cDNA was synthesized using LunaScript RT SuperMix Kit (New England Biolabs, #E3010, Hitchin, England), according to the manufacturer's instructions. RNA amplification was done using Luna Universal qPCR Master Mix (New England Biolabs, #M3003) and quantitative real‐time PCR was performed using Mx3005P Real‐Time PCR System (Agilent, Santa Clara, CA, USA). All experiments were performed in triplicate. The five benign control samples were pooled equally to create a pooled calibrator sample and utilized as the calibrator for all the qPCRs. Results were generated using the comparative Ct method [34] and were expressed as fold change relative to the pooled control calibrator sample. Primers for the qPCR were sourced from Sigma Aldrich (Gillingham, Dorset, UK).

### 2.4 Immuno‐Histochemistry (IHC)

To validate qPCR results for adiponectin receptors (ADIPOR1 and ADIPOR2), IHC was performed on FFPE blocks of 10 endometrial cancer tissues and 10 non‐cancer specimens. The antibodies used were: ADIPOR1 (Proteintech, Manchester, UK, #66619‐1‐Ig; anti‐mouse antibody) and ADIPOR2 (Abcam, Cambridge, UK, #ab223752; anti‐rabbit antibody). Human thyroid gland tissue was used as a positive control for ADIPOR1, and human placental tissue was used for ADIPOR2, both exhibiting cytosolic enhancements.

Tissue sections were processed, microwave antigen retrieval performed using a 0.01 M citrate buffer (pH 6.0), and thereafter blocking performed with 2.5% horse serum in PBS/BSA 1% (Vector Laboratories, #PK‐7200, Newark, CA, USA) and Avidin/Biotin (Vector Laboratories, #SP‐2001) and the slides were incubated overnight at 4°C with primary antibodies (ADIPOR1/ADIPOR2) diluted in PBS/1% BSA (ADIPOR1 1:50 dilution and ADIPOR2 at 1:40 dilution). Primary antibody incubation was followed by secondary antibody treatment (Vector Laboratories, #PK‐7200) and staining with 3,3′‐Diaminobenzidine DAB (Vector Laboratories, DAB Peroxidase Substrate kit, #SK‐4100). Counterstaining was done with Haematoxylin QS (Vector Laboratories, #H3404).

IHC scoring, ranging from ‘0’ to ‘2+’, was conducted by a single histopathologist blinded to the slides, where ‘0’ to ‘2+’ indicated gradually increasing cytosolic expression of the ADIPOR1/ADIPOR2 receptors.

### 2.5 Statistical Analysis

Statistical calculations were performed using Microsoft Excel, GraphPad Prism 9, G‐power 3.1.9.7 and R (version 4.3.1) software.

Logarithmic transformation [log_2_(expression+1)] was performed for all data to address skewness in distribution of results.

The biomarker expressions were correlated with different demographic characteristics of the cancer patients such as BMI, age, ethnicity, parity, use of HRT, use of contraception, diabetes, hypertension, history of cancer and family history. For ease of comparison, the populations were divided into binary groups for the calculations. BMI was categorised as normal weight and overweight/obese and age groups were divided into < 50 years and ≥ 50 years. 50 years was used as a cut‐off for age group as this is when most women typically enter menopause. Parity was divided into nulliparous (P0, patients who have never given birth) and multiparous (P0+, patients who have had more than or equal to one live birth). Initial calculations were performed using univariate linear regression and thereafter multivariate linear regression was performed using the significant associations from the univariate linear regression. All analyses were performed using functions ‘lm’ and ‘Anova’ from ‘stats’ and ‘car’ R packages, respectively.

Correlations between biomarker levels and tumor grade, stage, histology, LVSI, MELF and MSI among cancer patients were calculated using linear regression, univariate followed by multivariate, as applicable, as above. The endometrial cancer population was divided into pairs for easier comparison during calculations. Stages were categorized as Stage I (limited to the uterus) and stages II and III (involving spread beyond the uterus). While the histological classification of type 1 and type 2 are less commonly used today due to the emergence of molecular classification, all grade 3 endometrioid endometrial cancers were categorized as type 2 due to their significant similarities with type 2 tumors. Consequently, comparisons were made between grade 1 and grade 2 endometrioid endometrial cancers, both of which fall under the type 1 classification.

Paired *t*‐tests were utilized to compare expressions of the genes between different tissue types such as endometrial cancer tissue and adipose tissue in the same 39 patients and between endometrial cancer tissue and lymph nodes (*n* = 12).

The heatmaps were created using GraphPad Prism. The correlation matrices were built in GraphPad Prism using Pearson's correlation calculations.

Overall, a *p*‐value of less than 0.05 was considered significant. To account for multiple testing across the 10 genes examined, we applied the Benjamini–Hochberg procedure to control the false discovery rate (FDR) at 5%. Genes were considered statistically significant if their adjusted *p*‐values (*q*‐values) fell below the FDR threshold. All analyses were performed in RStudio.

A post hoc power analysis was conducted for three statistical tests using a significance level of 0.005 to account for multiple testing across 10 genes for a sample size of 39. For Pearson's correlation (large effect size, *r* = 0.5), power was 69%. For a paired *t*‐test (large effect size, *d* = 0.8), power was 97.3%. For a linear multiple regression (large effect size, *f* = 0.35), power was 62.7%. These results indicate sufficient power for the *t*‐test and moderate power for the correlation and multiple linear regression analyses for 39 samples. The power calculation for the 12 lymph node samples, however, was quite low to be of any substantial significance.

## 3 Results

### 3.1 Demographic Characteristics

The demographic characteristics of the 39 endometrial cancer patients included in this part of the study are illustrated in Table S1. The mean age of this population was 66.23 years (range 34–95 years), and their average BMI was 31.4 (range 20–53.2).

### 3.2 Endometrial Cancer Tissue Characterization in Study Patients

Most patients were diagnosed with grade 1 endometrial cancer (38.5%), followed by grades 2 (28.2%) and 3 (33.3%). Stage I cases predominated (79.5%) over Stage II/III (20.5%), based on FIGO 2009 classification. Histologically, following Bokhmann's classification [35] 66.7% were type 1 cancers, that is, endometrioid, while 33.3% were type 2 (e.g., grade 3 endometrioid, serous, mucinous, carcinosarcoma). Grade 3 endometrioid was classified as type 2 histology due to its closer clinical and immunohistochemical alignment with type 2 rather than type 1 cancers [36]. However, as previously mentioned, the terms type 1 and type 2 histology are used less frequently today due to the development of molecular classification. LVSI was found in 33.3%, MELF in 16%, and MSI in 21.9% of endometrial cancer. Data presented in Table S2. Further details are in a prior publication [28].

### 3.3 Expression of the Biomarkers and Their Receptors in the Endometrial Cancer Tissue (qRT‐PCR)

**FIGURE 1:**
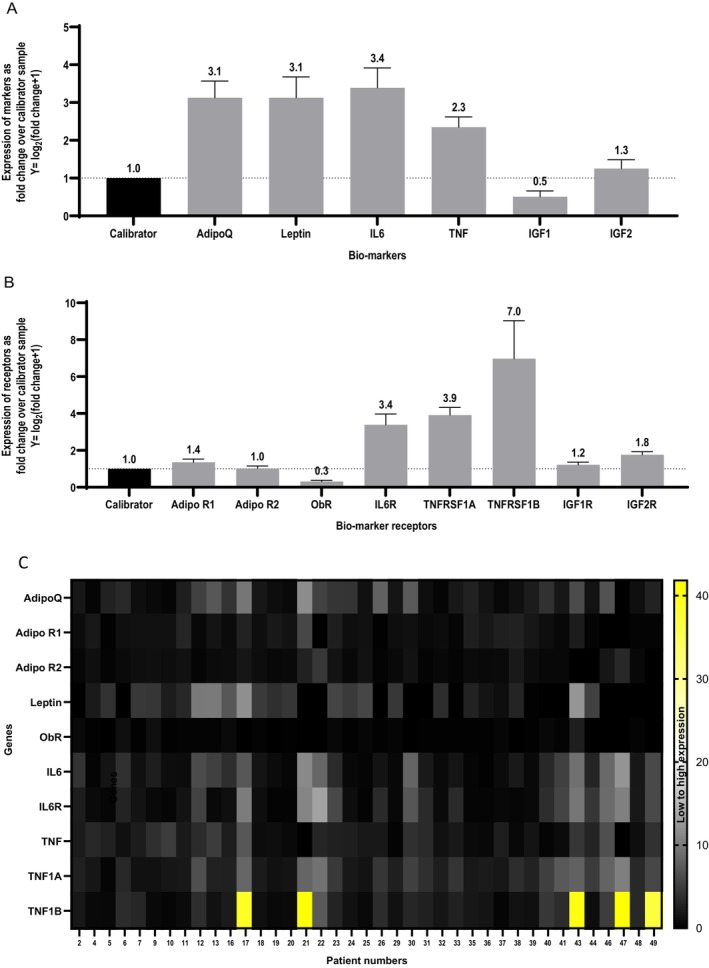
(A, B) Bar‐graph demonstrating the expression of (A) biomarkers and (B) their receptors in endometrial cancer tissue (*n* = 39). *X* axis‐ biomarkers (A), receptors (B). *Y* axis–expressions of biomarkers as fold change over benign endometrial calibrator sample (assigned value = 1, for comparison with the cancer samples). The error bars represent standard errors of mean. 1 (C) Heat map demonstrating the expression of all 6 biomarkers and their receptors in endometrial cancer tissues (*n* = 39). AdipoQ, adiponectin; ADIPOR1/R2, adiponectin receptors; OBR, leptin receptor; TNF1A, TNFRSF1A; TNF1B, TNFRSF1B (TNF receptors). Gene expressions are plotted on the *Y*‐axis against the individual patients on the *X*‐axis. Gene expressions are plotted as fold changes over a single pooled calibrator sample value. The color scale ranges from black (lowest value) to white (median) to yellow (highest value).

The mRNA expressions of adiponectin (ADIPOQ), leptin, IL6 and TNFα and their receptors (ADIPOR1, ADIPOR2, OBR, IL6R, TNFRSF1A, TNFRSF1B) were studied in the endometrial cancer tissue using qRT‐PCR and fold change relative to the calibrator benign endometrial control sample were calculated, as outlined in the materials and methods section. In comparison to normal endometrium used as a reference or calibrator, the expressions of all the adipocytokines—adiponectin, leptin, IL6, and TNFα—were found to be much higher in endometrial cancer tissue (Figure 1A).

Concerning the receptors, IL6R and both TNF receptors were markedly upregulated in endometrial cancer tissue compared to the control sample. In contrast, ADIPOR1 was mildly over‐expressed in the cancer tissue, whereas ADIPOR2 showed similar expression to the control sample. Contrarily, the expression of leptin receptor (OBR) was reduced by about two‐thirds in the cancer tissue compared to the control population (Figure 1B).

To appreciate the individual differences of expression of these markers and their receptors, this data is also expressed in the form of a heatmap (Figure 1C).

Thereafter, we correlated the markers and their receptor expressions in endometrial cancer tissue using Pearson's correlations. A strong correlation between the expression levels of adiponectin and IL6 was noted (Figure S1), that suggest that these markers may have similar regulatory mechanisms and work via interconnected pathways such as MAPK, PI3K/Akt/mTOR and MEK/ERK, indicating their joint involvement in endometrial cancer development.

### 3.4 Association Between Biomarker Expression and Patient Demographics and Tumor Characteristics

Associations were examined between biomarker mRNA expression in the endometrial cancer tissues and patient demographics and tumor characteristics using linear regression. Among the significant associations noted between biomarker expression and different patient demographics, a higher ADIPOR2 expression was noted in patients of Asian ethnicity (*p* = 0.043) and a higher TNF level was noted in patients on HRT (*p* = 0.039) (Figure S2A).

ADIPOR1 was significantly lower in patients without LVSI (*p* = 0.007), and leptin was significantly higher in patients with grade 2 endometrial cancer than grade 1 (*p* = 0.045) (Figure S2B).

Interestingly, the associations between the two adiponectin receptors and endometrial cancer characteristics displayed opposing patterns (Table 1). ADIPOR1 expression was elevated in grade 1 type 1 endometrial cancer, while ADIPOR2 was higher in type 2 cancers and showed similar levels in grade 1 and 2 cancers. ADIPOR1 expression was lower in the presence of LVSI, MELF pattern, and MSI, whereas ADIPOR2 expression was higher with LVSI and the MELF pattern. Although not all associations reached statistical significance, the contrasting trends are noteworthy.

**TABLE 1:** Associations between Adiponectin receptors (ADIPOR1 and ADIPOR2) and cancer characteristics are plotted using Least Square means (LS means).

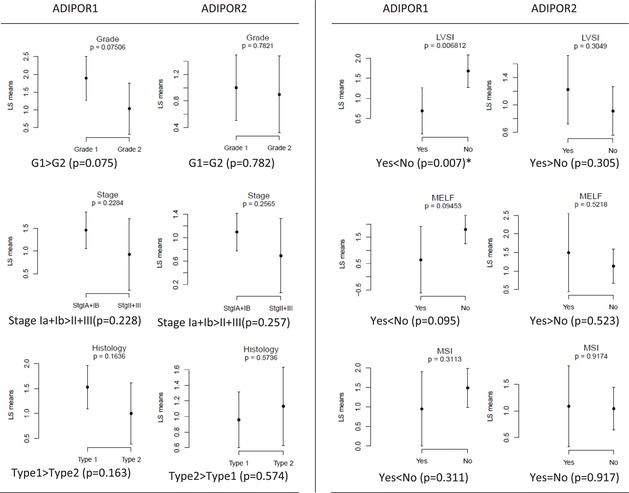

*Note:* X axis‐cancer characteristics' groups; Y axis‐log_2_ [(Fold change/expression of marker) +1]. The dots represent the LS mean values, and the vertical lines represent 95% confidence intervals.

### 3.5 Expression of the Biomarkers and Their Receptors in the Adipose Tissue and in Lymph Nodes (qRT‐PCR)

**FIGURE 2:**
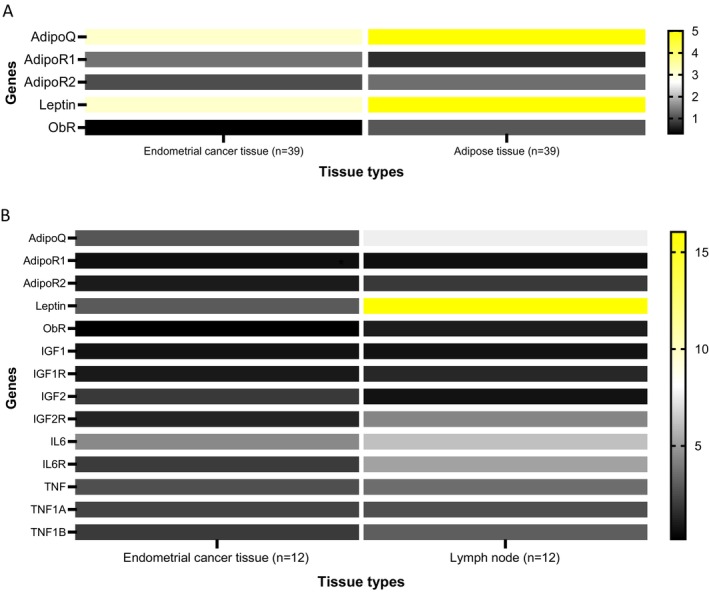
Heat map comparing the expression of (A) adiponectin, leptin and their receptors in endometrial cancer tissue and parametrial adipose tissue (*n* = 39) (B) all 6 biomarkers and their receptors in endometrial cancer tissue and lymph nodes (*n* = 12). AdipoQ, adiponectin; AdipoR1/R2, adiponectin receptors; ObR, leptin receptor; TNF1A, TNFRSF1A; TNF1B, TNFRSF1B (TNF receptors). Gene expressions are plotted on the *Y*‐axis against the individual patients on the *X*‐axis. Gene expression plotted as fold changes over a single pooled calibrator sample. The color scale ranges from black (lowest value) to white (median) to yellow (highest value).

The mRNA expression of adiponectin, leptin, and their receptors was analyzed in the adipose tissue of 39 patients with fresh endometrial cancer samples. Adipose tissue showed significantly higher levels of adiponectin, leptin, and leptin receptor (*p* < 0.0001, *p* = 0.0026, *p* < 0.0001, respectively) compared to endometrial cancer tissue, while ADIPOR1 and ADIPOR2 expression showed no significant differences (Figure 2A).

In 12 lymph nodes (4 cancerous and 8 non‐cancerous), significantly higher adiponectin (*p* = 0.0002), leptin (*p* = 0.011), and IL6R (*p* = 0.009) expression was observed compared to endometrial cancer tissue. Among these, three of four cancerous lymph nodes and five of eight non‐cancerous nodes were associated with LVSI in the endometrial tissue (Figure 2B).

Comparison of expression of the biomarkers between three tissue types has been presented in the Figures S3–S5.

### 3.6 Association Between Biomarker and Their Receptor Expression in Adipose Tissue/Lymph Nodal Tissue and Tumor Characteristics

No significant correlations were found between expression of the biomarkers or their receptors in adipose tissue and endometrial tumor characteristics.

**FIGURE 3:**
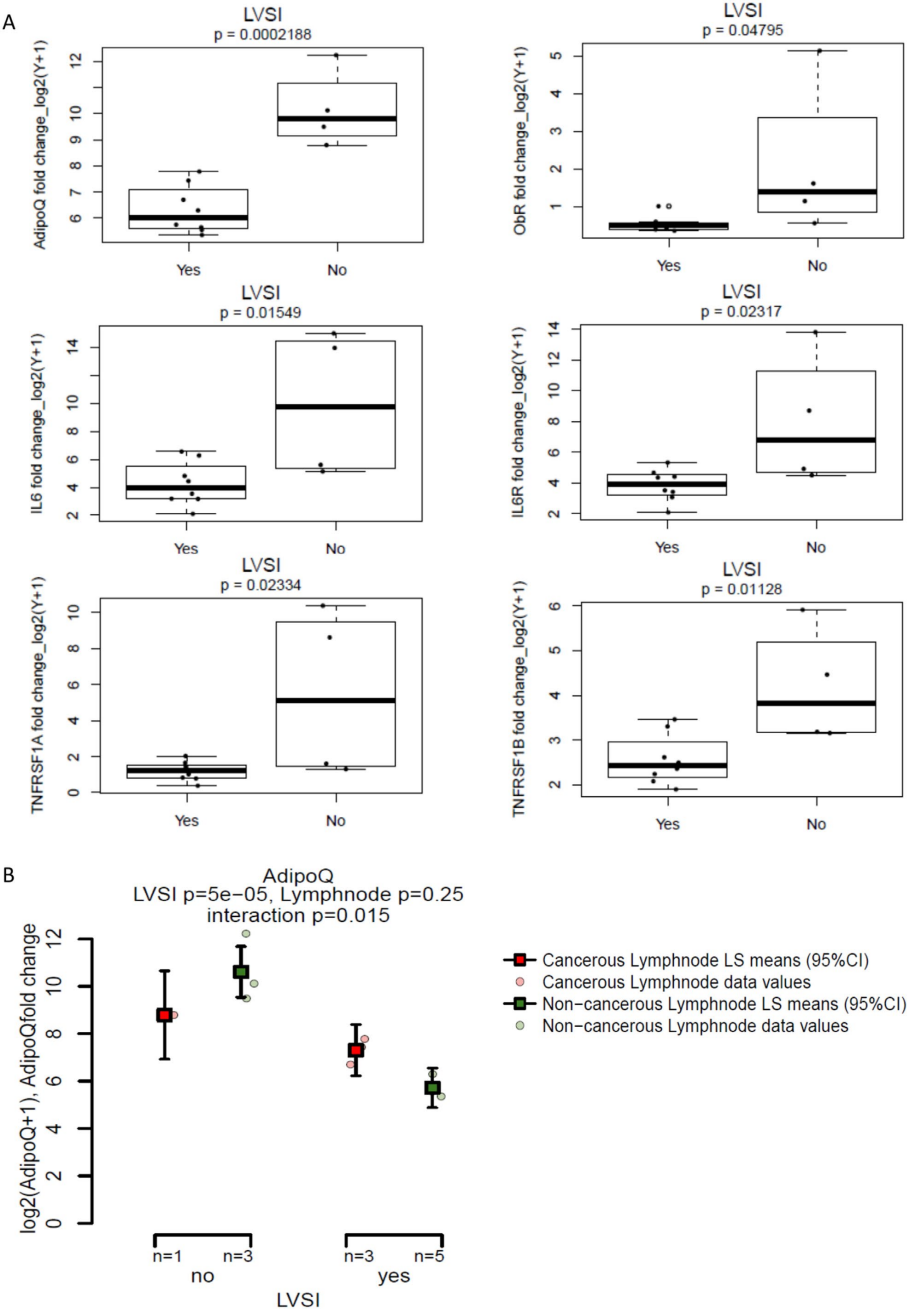
(A) Box plots to demonstrate the association between biomarker or their receptor expression in lymph nodes and LVSI, using univariate linear regression (*n* = 12). *X* axis‐ prognostic factors groups; *Y* axis‐ log2 [(Fold change/expression of marker) +1]. The 25th and 75th percentiles are represented by the lower and upper boundaries of the rectangles, respectively, while the median is indicated by the horizontal line inside the rectangles. The whiskers extend from the box denoting the minimum and maximum values. Results represent univariate analysis only, as no other associations reached statistical significance to necessitate a multivariate analysis. 3 (B) Two‐way ANOVA demonstrating the connection between adiponectin in cancer positive and negative lymph nodes and comparison with LVSI in endometrial cancer tissue. ADIPOQ, adiponectin; Y, LVSI present in the endometrial cancer tissues; *N* = no LVSI noted in the cancer tissues. In patients without LVSI, adiponectin was significantly higher in cancer negative lymph nodes but in patients with LVSI, adiponectin was significantly higher in the cancer positive lymph nodes.

Considering the expression of biomarkers in lymph nodes, the presence or absence of LVSI in the endometrial cancer tissue was noted to have significant associations with the expression of certain markers in the lymph nodes, which have been demonstrated using box plots (Figure 3A). Adiponectin (*p* = 0.0002), OBR (*p* = 0.048), IL6 (*p* = 0.015), IL6R (*p* = 0.023), TNFRSF1A (*p* = 0.033), and TNFRSF1B (*p* = 0.011) had reduced levels of expression in the lymph nodes in patients with LVSI. These correlations indicate that the presence of LVSI could impact the expression of inflammatory markers in the lymph nodes, especially adiponectin. No other significant associations were noted between tumor characteristics and biomarker expression in lymph nodes.

Thereafter, considering spread of cancer to lymph nodes, we compared the four cancerous lymph nodes to eight non‐cancerous lymph nodes, no significant differences were found in the expression of any of the markers studied within the limitations of a small sample size. However, an interesting statistical interaction was noted between LVSI in the endometrial cancer tissue, cancer status of lymph node and adiponectin, using a linear model of ANOVA (Figure 3B). In patients without LVSI (*n* = 4), adiponectin expression was calculated to be significantly higher in cancer‐negative lymph nodes whereas, in patients with LVSI (*n* = 8), adiponectin was significantly higher in the cancer‐positive lymph nodes. However, despite the statistical significance, the limited number of lymph node studied means that any clinical relevance must be confirmed through larger studies.

### 3.7 Comparison Between Tissues Expression and Circulating Levels of the Biomarkers

The expression of adipocytokine in tissues was compared with its levels in plasma to assess any correlation (Figure S6A). Detailed assessments of the plasma levels for these markers have been previously published [28]. Adiponectin expression was higher in endometrial cancer tissue compared to control sample. However, in plasma, adiponectin levels were significantly lower in patients with endometrial cancer. Leptin levels were higher in endometrial cancer tissue compared to control endometrium, but unlike adiponectin, leptin levels in plasma were also elevated in the cancer patients. IL6 and TNF are expressed more in the cancer tissue than in the control endometrium which is opposite of what is seen in plasma samples, where both IL6 and TNF are higher in the control subjects.

No significant correlations were identified between plasma levels of adiponectin, leptin, IL6 and TNF and their expression in endometrial cancer tissue or their receptor levels in the same tissue (Figure S6B,C). Thus, it appears from this calculation that the levels of adipocytokines in endometrial cancer tissues and plasma are independent of each other, suggesting that the tumor possibly does not release adipocytokines into circulation, nor do circulatory levels influence their expression in tissues or their receptors.

### 3.8 Immunohistochemistry (IHC)

**FIGURE 4:**
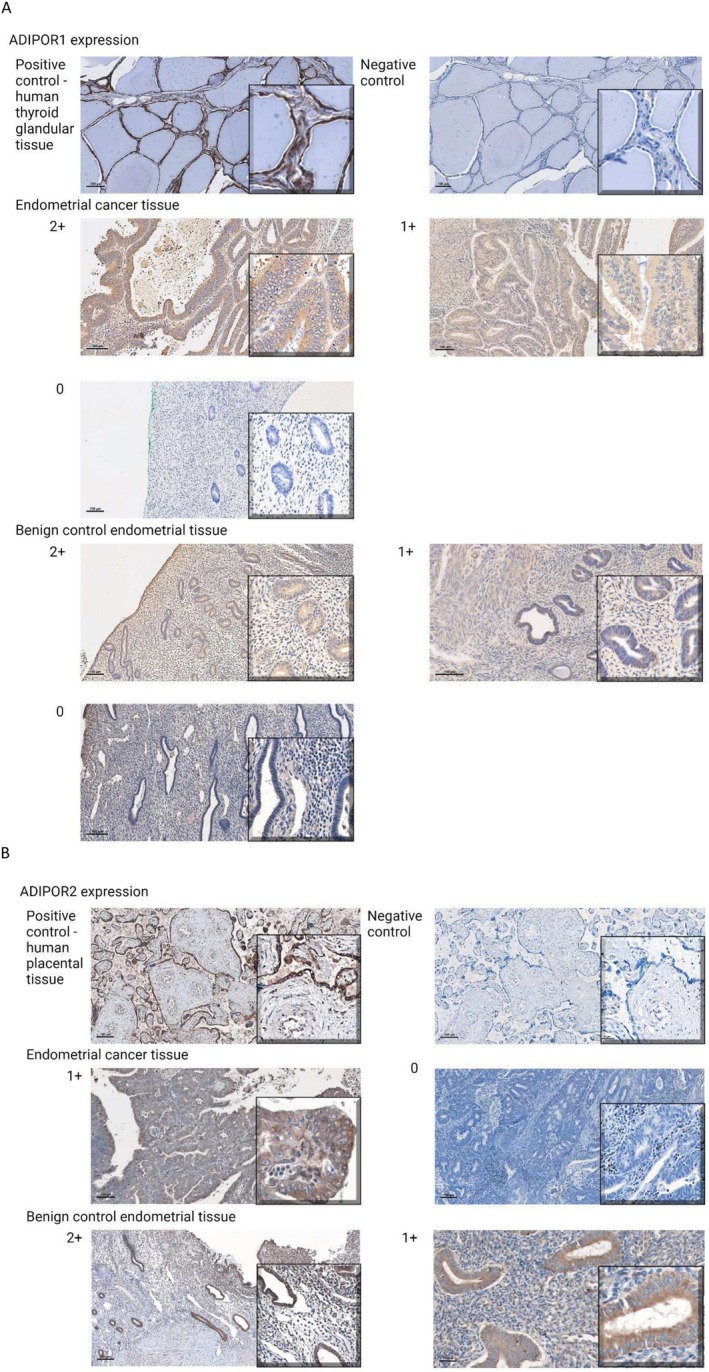
Illustration of chromogenic 3,3′‐Diaminobenzidine (DAB) staining for ADIPOR1 (A) and ADIPOR2 (B) receptor in endometrial cancer tissue and benign tissues. Image taken at 10× magnification, Scalebar = 100 μm.

IHC was carried out from FFPE blocks of 10 endometrial cancer tissue (from among the study population) and 10 non‐cancer specimens (from among the control population). Examples of ‘0’ to ‘2+’ expression of the two adiponectin receptors ADIPOR1 and ADIPOR2 have been illustrated in Figure 4.

We observed a heightened expression of ADIPOR1 in endometrial cancer tissue compared to the control benign endometrium (Table 2). Conversely, the same cancer tissue exhibited lower expression of ADIPOR2 than the same benign control endometrium (Table 2). No association were noted between either adiponectin receptor expression and BMI or tumor characteristics (*p* < 0.05, figures not presented as no associations noted).

**TABLE 2:** Table illustrating the expression of ADIPOR1 and ADIPOR2 in endometrial cancer tissue (serial no. EC1‐10) and benign endometrial tissue (serial no. C1‐10) using IHC.

Endometrial cancer tissue (serial no.)	ADIPOR1	ADIPOR2	Benign endometrial tissue (serial no.)	ADIPOR1	ADIPOR2
EC1	1+	1+	C1	0	1+
EC2	2+	1+	C2	2+	1+
EC3	0–1+	1+	C3	0–1+	2+
EC4	0	0	C4	0	1+
EC5	0–1+	1+	C5	0	1+
EC6	1+	1+	C6	0	1+
EC7	1+	1+	C7	0	1+
EC8	2+	0	C8	0	1+
EC9	0	0	C9	1+	1+
EC10	0–1+	0	C10	0	2+

IHC results and qPCR results from the same tumor tissues did not correlate strongly with each other, implying the need for large scale IHC to validate or refute mRNA expression quantified via qPCR. Validation of the IHC results and comparison between PCR and IHC results have been presented in the Figures S7–S9.

## 4 Discussion

Recent large‐scale epidemiological studies have established a clear connection between obesity and an elevated risk of endometrial cancer [37, 38]. However, this association may not be entirely influenced by the stage, grade, or even the histological classification of endometrial cancer [39, 40]. Biological and molecular pathways linked to obesity, such as via alteration of adipocytokines play a role in tumor development by promoting uncontrolled cell proliferation and new blood vessel formation, which aids metastasis [41]. Moreover, as the tumor progresses, tissue hypoxia further regulates the expression of growth factors and their receptors, aiding cancer advancement [42].

The results of this study provide evidence that adipocytokines and their receptors play a critical role in the pathophysiology of endometrial cancer, demonstrating distinct expression patterns that differentiate cancerous tissue from benign endometrium. The increased mRNA expression of adiponectin (ADIPOQ), leptin, IL6, and TNFα in endometrial cancer tissue highlights an enhanced inflammatory and metabolic state within the tumor microenvironment, which may drive cancer progression. The marked upregulation of IL6R and TNF receptors (TNFRSF1A, TNFRSF1B) in cancer tissue compared to controls supports the notion that these cytokines and their signaling pathways are central to the development and aggressiveness of endometrial cancer. There is limited literature on the expression of these markers and their receptors in endometrial cancer. Takemura et al. demonstrated adiponectin and ADIPOR1/ADIPOR2 expression in human endometrium, noting increased expression during early pregnancy [43]. Subsequent studies by Yamauchi et al. [44] and Yabushita et al. [45] investigated their expression in endometrial cancer tissue, with Yabushita reporting receptor expression in 60% of samples. Our study also noted a differential expression of adiponectin receptors (ADIPOR1 and ADIPOR2) which is particularly noteworthy. ADIPOR1 was mildly overexpressed in cancer tissue, suggesting a role in the altered metabolic signaling in the tumor, while ADIPOR2 remained unchanged, possibly indicating a more stable or non‐dominant role in this context.

The correlations between mRNA expression levels of adipocytokines in cancer tissue and patient demographics or tumor characteristics reveal important associations that could influence prognosis and could also act as therapeutic targets. The opposite associations of ADIPOR1 and ADIPOR2 with grade, histology, LVSI, and MELF underscore the complexity of these receptors' roles in endometrial cancer, warranting further exploration of their opposing functional implications. Being primarily expressed in skeletal muscle, ADIPOR1 mainly binds globular adiponectin and increases fatty acid oxidation and glucose uptake in skeletal muscle by the AMPK and APPL‐1 pathways [46]. Whereas ADIPOR2 is primarily found in liver, mainly binding full length adiponectin and promoting hepatic glucose production and lipid metabolism by activating the AMPK pathway [46]. While both receptors share 67% amino‐acid homology and overlapping functions, they also exhibit tissue‐specific roles and signalling pathways. Yabushita et al. reported that the expression of ADIPOR1 inversely correlated with high histological grade, deep myometrial invasion, LVSI, adnexal invasion, and lymph node metastasis whereas ADIPOR2 expression was not related to any prognostic factors supporting a differential profile of ADIPOR1 and ADIPOR2 [45]. However, to the best of our knowledge, our study is the first to report this opposite association of ADIPOR1 and ADIPOR2 with grade, histology, LVSI, and MELF. Yabushita et al. suggested that upregulating ADIPOR1 or developing specific agonists (osmotin/PPARα agonist/ADIPORon) may prove beneficial in treating endometrioid adenocarcinomas [45]. However, with the differential expression we noticed, it remains to be seen if downregulating ADIPOR2 at the same time leads to any added treatment benefit. Needless to say, the relationship between adiponectin and its receptors is complex and multifaceted and understanding their distinct metabolic effects is crucial for developing targeted therapies.

Exploring the prognostic significance of adiponectin and leptin receptors in endometrial cancer, Busch et al. reported no significant associations between the receptor expressions and clinicopathological characteristics [8]. In our study, LVSI was the only prognostic factor found to be associated with biomarker expression, showing significant correlations with ADIPOR1, ADIPOR2, OBR, and IL6R. Watanabe et al. previously suggested that cell cycle‐related genes might play a role in the development of LVSI in endometrial cancer [47]. Since adipocytokines are known to influence cell cycle regulation [20], these connections could explain the observed associations with LVSI. Validating these molecular markers could improve the accuracy of LVSI diagnosis and guide treatment decisions, especially in predicting LVSI from endometrial biopsies before surgery, potentially supporting the use of sentinel lymphadenectomy in low‐risk patients.

The absence of correlation between tissue and plasma levels of adipocytokines, as well as between tissue receptor levels and plasma adipocytokine levels, suggests that these biomarkers may primarily act within the local tumor microenvironment rather than serving as systemic indicators of disease. Yabushita et al. [45] also noted a lack of correlation between tissue receptor levels and plasma levels of adiponectin and leptin. However, to our knowledge, no other study has previously examined the relationship between plasma and tissue levels of all four adipocytokines. This discrepancy underscores the importance of conducting tissue‐specific analyses when investigating the role of adipocytokines in cancer and highlights the need to be cautious about relying solely on plasma levels for prognostic or diagnostic purposes. However, the study's small sample size may have limited its power to detect a significant difference. Additionally, correcting for multiple comparisons across numerous tests could further weaken the observed correlations between tissue and circulatory marker levels.

The significant differences in adipocytokine and receptor expression between endometrial cancer tissue and adipose tissue, as well as the distinct expression profiles observed in sentinel lymph nodes with and without cancer, underscore the potential utility of these biomarkers in differentiating between cancerous and non‐cancerous tissues. To our knowledge, this is the first study examining adiponectin, leptin, and their receptors in parametrial adipose tissue and lymph nodes in endometrial cancer. The finding that higher expression of adiponectin and leptin was noted in adipose tissue compared to endometrial cancer tissue suggests their significant role in regulating adipose tissue functions and metabolism. However, the difference could also be exaggerated as we used endometrium as the calibrator sample instead of adipose tissue from control patients. Also, while adiponectin receptor expression showed no notable differences between adipose and cancer tissues, leptin receptor was significantly higher in adipose tissue. This implies that the adiponectin receptors are possibly expressed uniformly across various tissues and cell types, indicating their widespread presence and potential involvement in diverse physiological processes involved in glucose and lipid metabolism in different target tissues such as adipose tissue, skeletal muscle, and liver [48]. A high leptin receptor expression has been reported in adipose tissue, highlighting the role of leptin in both autocrine and paracrine signaling modulation in the adipose tissue. High leptin receptor expression in adipose tissue facilitates communication between adipocytes and the nervous system, influencing feeding behavior and energy expenditure [49].

In the context of endometrial cancer, the high expression of leptin receptors in adipose tissue may enhance leptin‐driven lipolysis, alongside the influence of cancer cells on cancer‐associated adipocytes [50]. This could create a tumor‐supportive niche that fosters cancer cell growth, metastasis, and overall disease progression. Conversely due to its involvement in cancer progression, targeting the leptin receptors in the adipocytes could potentially prevent disease advancement. In the era of personalized cancer therapy, there remains a significant unmet need for developing targeted therapeutic strategies specifically for patients with both obesity and cancer or pre‐cancer [50].

Thereafter, we also report the expression of our markers and their receptors in 12 lymph node samples from patients who underwent sentinel lymphadenectomy. An interesting finding was altered expression of various markers in lymph nodes relative to LVSI in cancer tissue. Patients with LVSI demonstrated reduced expression of several biomarkers and their receptors in lymph nodes, suggesting interactions between LVSI and inflammatory pathways. This also highlights the complex association between LVSI and the spread of cancer to lymph nodes [51].

The finding that adiponectin expression was higher in lymph nodes with cancer metastasis, particularly in the presence of LVSI, suggests that adiponectin might serve as a marker for metastatic potential in endometrial cancer. This suggests that adiponectin may play opposing roles in cancer metastasis to lymph nodes depending on the presence or absence of LVSI in the cancer tissue—adiponectin preventing cancer metastasis to lymph nodes in patients without LVSI but promoting cancer metastasis to lymph nodes in patients with LVSI. It has been noted in reviews discussing contentious aspects of adiponectin that it may be regulated in the opposite direction and may even exert a pro‐inflammatory effect in obesity‐independent classic inflammatory conditions [52]. Adiponectin has been reported to play a dual role in inflammation, exhibiting both pro‐ and anti‐inflammatory effects [53]. This may be attributed to its various isoforms, such as on one hand the full‐length adiponectin suppressing inflammation via PI3K‐mediated pathways, promoting macrophage migration, while on the other hand the globular adiponectin (gAd) activates NF‐κB signaling, enhancing pro‐inflammatory cytokine production [54]. These pathways also involved differential association with the adiponectin receptors [55]. Studies have also reported that adiponectin's effects on macrophages may vary based on stimulation duration, initially inducing inflammatory TNF‐α production, which subsequently increases IL‐10 expression, triggers autophagy, and reduces LPS‐mediated inflammation [56]. Hence, it is plausible that within this altered immune milieu of the tumor microenvironment, altered adiponectin expression may even favor tumor progression and metastasis. However, the small number of patients in each category limits the wider extrapolation of this finding. Nevertheless, the finding could have implications for understanding the interaction between adiponectin and LVSI and the role of adiponectin in the spread of cancer to lymph nodes. This warrants further investigation to better comprehend its significance in different subgroups of patients based on LVSI status. Furthermore, there was a lack of any significant correlation between biomarker and receptor expression in endometrial tissue and lymph nodes, suggesting that these markers may not predict microscopic cancer spread to lymph nodes. However, again this lack of correlation may be due to the sample size being too small to detect a meaningful difference.

The immunohistochemistry (IHC) results provide an additional layer of complexity, revealing inconsistencies between mRNA expression and protein levels of adiponectin receptors in cancer tissues. This finding suggests that post‐transcriptional modifications, protein stability, or other regulatory mechanisms may influence receptor expression in ways not captured by mRNA analysis alone. The small sample size of the IHC analysis limits the broader applicability of our results; therefore, larger‐scale IHC studies are essential to validate these findings and facilitate the translation of molecular insights into clinical practice.

While our study comprehensively analyzed markers and their receptors in anatomically related tissues, certain limitations exist. Due to shortage of benign endometrial samples, the study used five benign endometrial control samples, which were pooled to create a single calibrator sample for all qRT‐PCR comparisons. This approach, while practical, can potentially mask the individual variability between control samples, which might obscure biological variability and influence the accuracy of fold change calculations. Also, the lack of matched control samples led to the use of benign endometrial tissue as a calibrator for all fold change calculations. While this approach provided a standardized basis for comparing expression levels with endometrial cancer tissues, using endometrial tissue as the calibrator for adipose tissue and lymph nodes may have led to an overestimation of marker expression in these tissues. This is particularly relevant for adipocytokines, such as leptin and its receptors, which are naturally more abundant in adipose tissues. To improve accuracy in future studies, it is essential to obtain appropriate control samples for adipose tissue and lymph nodes to ensure more precise comparisons and avoid potential biases in expression analysis. Also, adipose tissue from different sites of body could be sampled to assess the relative differences in contribution between visceral and sub‐cutaneous adipose tissue in adipocytokine secretion. Additionally, the limited sample size for lymph nodes might affect the reliability of findings by reducing the statistical power, limiting the ability to detect meaningful associations or validate observed trends such as the significant correlations found between LVSI and biomarker expression in lymph nodes. The lack of difference in expression of various markers between cancerous and non‐cancerous lymph nodes could be due to the sample size being too small to detect subtle or moderate effects. Further, protein expression evaluation for all the markers and their receptors was lacking. qPCR estimates the mRNA expression of the markers; however, mRNA expression does not always translate to protein synthesis. It would be an interesting addition to our results if protein expression could be analyzed for all the markers using either IHC or multiplex technologies. IHC analysis of a larger, matched number of markers and their receptors, combined with a multiple biomarker panel study such as using multiplex, might be particularly helpful in this situation. The IHC results may also be limited by potential subjectivity due to use of a single pathologist, especially without digital image analysis or validation by multiple pathologists. Lastly, the study predates the newer molecular classification of endometrial cancer. Hence, we were unable to correlate the circulating and tissue levels of the markers with POLE and p53 mutation testing. Although we had MSI results for some patients and found no significant association with the markers, this analysis was not performed for all patients, indicating that further investigation is needed in this respect.

### 4.1 Clinical Implications of the Research

The aim of this study was to evaluate the prognostic value of the selected adipocytokines and their receptors, and the following clinical implications could be derived from the study:

- Prognostic Biomarkers for Risk Stratification: ADIPOR1 was associated with less aggressive, well‐differentiated grade 1/type 1 cancers and favorable prognostic factors, suggesting its potential role as a marker for low‐risk disease. ADIPOR2, on the other hand, was linked to type 2 cancers, LVSI, and MELF, indicating a role in more aggressive tumors with poorer prognosis. This divergent expression pattern could help refine molecular subtyping in endometrial cancer, aiding in personalized treatment planning.
- Enhancing Diagnostic Accuracy for LVSI and Pre‐Surgical Assessment: Significant associations were noted between AdipoR1, ObR, IL6R, and IGF2 with LVSI. If validated, these biomarkers could be incorporated into pre‐surgical endometrial biopsies, helping to detect LVSI early and hence indicating which low‐risk patients will benefit from sentinel lymph node dissection.
- Potential Therapeutic Targets: The distinct expressions of ADIPOR1 and ADIPOR2 in endometrial cancer tissues and increased expression of leptin receptor in adipose tissue suggest that they have potential for use as targeted therapy. Personalized treatment strategies could be developed to modulate adiponectin and leptin receptor pathways, particularly in obesity‐associated endometrial cancer.

To fully integrate these biomarkers into clinical practice, larger prospective studies and clinical validation are necessary to confirm their diagnostic, prognostic, and therapeutic potential.

## 5 Conclusion

This study underscores the significant role of adipocytokines and their receptors in the molecular landscape of endometrial cancer, highlighting their involvement in inflammatory and metabolic pathways that may drive tumor progression. The strong correlations observed between specific adipocytokines and patient or tumor characteristics suggest potential avenues for personalized therapeutic strategies, particularly in considering ethnic and hormonal influences on biomarker expression.

The lack of correlation between plasma and tissue levels of adipocytokines emphasizes the importance of tissue‐specific biomarker assessment in understanding tumor biology and cautions against the use of plasma markers alone for clinical decision‐making. Furthermore, the discrepancies between mRNA and protein expression levels in cancer tissues call for more comprehensive studies to elucidate the regulatory mechanisms governing adipocytokine receptor expression in endometrial cancer.

Overall, this study contributes valuable insights into the complex roles of adipocytokines in endometrial cancer, pointing to the need for further research to fully unravel their potential as biomarkers and therapeutic targets. The observed associations with LVSI and metastatic potential, particularly concerning adiponectin, open new avenues for exploring the prognostic significance of these markers in endometrial cancer progression and metastasis.

## Author Contributions

I.R., A.M., P.E.E., and L.B.M. made substantial contributions to the conception or design of this research project. I.R. recruited patients, collected samples, performed experiments, acquired and interpreted data, and drafted the original manuscript. C.S.M.‐L. wrote the codes for the statistical analyses. A.M. reviewed and edited the manuscript. S.B.‐M., J.C., and A.T. provided samples, reviewed the manuscript, and provided valuable input. B.H. provided the tissue samples, reviewed the histology reports and the manuscript, I.B. provided the tissue samples, performed the IHC scoring and reviewed the manuscript. L.B.M. analyzed and interpreted data and substantively revised the manuscript. P.E.E. provided samples and comprehensively reviewed and edited the manuscript. All authors have read and agreed to the published version of the manuscript.

## Ethics Statement

The study was conducted according to the guidelines of the Declaration of Helsinki and approved by the National Health Service Research Ethics Committee, United Kingdom—Health Research Authority and Health and Care Research Wales (IRAS Project ID: 285863).

## Consent

All the participants signed an informed consent form before participating in the study.

## Conflicts of Interest

The authors declare no conflicts of interest.

## Supporting information


Data S1.

## Data Availability

The raw data for analyses is not publicly available to preserve individuals’ privacy under the European General Data Protection Regulation.
