# Supplementary material for: Exploring the Relationship Between Adipocytokines and Endometrial Cancer: Identifying Correlations With Clinico‐Pathological Prognostic Factors
Source: Cancer Med. 2025 Jul 11;14(13):e71007. doi: 10.1002/cam4.71007 (PMC12246795; doi:10.1002/cam4.71007)
Supplement: Supplementary file 1 — Data S1. [file CAM4-14-e71007-s001.docx]

## Demographic characteristics

The demographic characteristics of the thirty-nine endometrial cancer patients included in this part of the study are illustrated in Supplementary Table 1. The mean age of this population was 66.23 years (range 34-95 years) and their average BMI was 31.4 (range 20-53.2).

| **Parameters** | | **Study population (n=39)** | |
| --- | --- | --- | --- |
|  |  | **Number** | **Percentage** |
| **Age range (years)** | <50 years | 4 | 10.3 |
|  | ≥50 years | 35 | 89.7 |
| **Parity**^1^ | P0 | 8 | 20.5 |
|  | P0+ | 31 | 79.5 |
| **BMI**^2^ | 18.5-24.9 (normal weight) | 9 | 23.1 |
|  | ≥25.0 (overweight/ obese) | 30 | 76.9 |
| **Ethnicity** | Caucasian | 32 | 82.1 |
|  | Asian | 7 | 17.9 |
| **Smoking status** | Yes | 14 | 35.9 |
|  | No | 25 | 64.1 |
| **Menopausal status** | Yes | 31 | 79.5 |
|  | No | 8 | 20.5 |
| **Hormonal Contraception** | Yes | 23 | 59 |
|  | No | 16 | 41 |
| **HRT**^3,4^  (menopausal women, n=31) | Yes | 9 | 29 |
|  | No | 22 | 71 |
| **Diabetes** | Yes | 10 | 25.6 |
|  | No | 29 | 74.4 |
| **Hypertension** | Yes | 18 | 46.2 |
|  | No | 21 | 53.8 |
| **Past h/o**^5^ **cancer** | Yes | 5 | 12.8 |
|  | No | 34 | 87.2 |
| **Family h/o**^5^ **cancer** | Yes | 18 | 46.2 |
|  | No | 21 | 53.8 |

Supplementary Table 1 : Demographic characteristics of the endometrial cancer patients (n=39).

Parity indicates the number of times a woman has given birth after 24 weeks of gestation, with nulliparous meaning no live births. BMI (Body Mass Index) is calculated as BMI = (weight in kg) / (height in m²). HRT refers to Hormone Replacement Therapy.

## Endometrial cancer tissue characterisation in study patients

| **Parameter** | **Sub-groups** | **N (%)** |  | **Parameter** | **Sub-groups** | **N (%)** |
| --- | --- | --- | --- | --- | --- | --- |
| **Grade** | 1 | 15 (38.5) |  | **LVSI** | Yes | 13 (33.3) |
|  | 2 | 11 (28.2) |  |  | No | 26 (66.7) |
|  | 3 | 13 (33.3) |  |  |  |  |
| **Stage** | IA + IB | 31 (79.5) |  | **MELF** | Yes | 4 (16) |
|  | II + III | 8 (20.5) |  | (reported n=25) | No | 21 (84) |
| **Histology** | Type 1 | 26 (66.7) |  | **MSI** | Yes | 7 (21.9) |
|  | Type 2 | 13 (33.3) |  | (reported n=32) | No | 25 (78.1) |

Supplementary Table 2: Distribution of endometrial cancer characteristics among study patients, categorized by grade, stage, histology, LVSI, MELF, and MSI.

LVSI refers to lympho-vascular space invasion, MELF to the microcystic, elongated, and fragmented pattern of invasion, and MSI to microsatellite instability.

1. **Correlation plots between biomarker and their receptor expression in endometrial cancer tissue (EC)**

To explore potential correlations, Pearson correlation analyses were conducted to examine the relationships between the expression levels of markers within endometrial cancer tissue and between the markers and their corresponding receptors in the same tissue (Supplementary Figure 1). A moderate correlation was observed between adiponectin and IL6 expression (r=0.6, p=0.013). On the other hand, there appears to be no significant correlation between the expression of leptin or TNF with the other genes. There is a low or almost negligible correlation observed between the expression levels of genes and their corresponding receptors, except for IL6 and IL6R, which exhibit a high correlation (r=0.9, p=0.000). Also noted was a high correlation between IL6 receptor IL6R and one of the TNF receptors, TNFRSF1A (r=0.9, p=000) and moderate correlations between IL6R and the other TNF receptor, TNFRSF1B (r=0.7, p=0.006) and in between the two TNF receptors, TNFRSF1A and TNFRSF1B (r=0.7, p=0.007).

The moderate correlations between adiponectin and IL6 suggest that these markers may have similar regulatory mechanisms and work via interconnected pathways such as MAPK, PI3K/Akt/mTOR and MEK/ERK, indicating their joint involvement in endometrial cancer development. Conversely a lack of correlation between the expression of leptin or TNF with the other genes suggest that these genes may be regulated independently from the expression of the other genes in this context.

Additionally, the strong correlation observed between IL6 and IL6R, along with the moderate to strong positive correlations detected between IL6 receptor and the TNF receptors, as well as between the two TNF receptors themselves, suggest a potential interplay among these molecules. The robust correlations suggest that the IL6 and TNF receptor expressions are possibly closely regulated and likely involved in a coordinated signalling pathways such as the JAK/STAT3 and MAPK pathways.


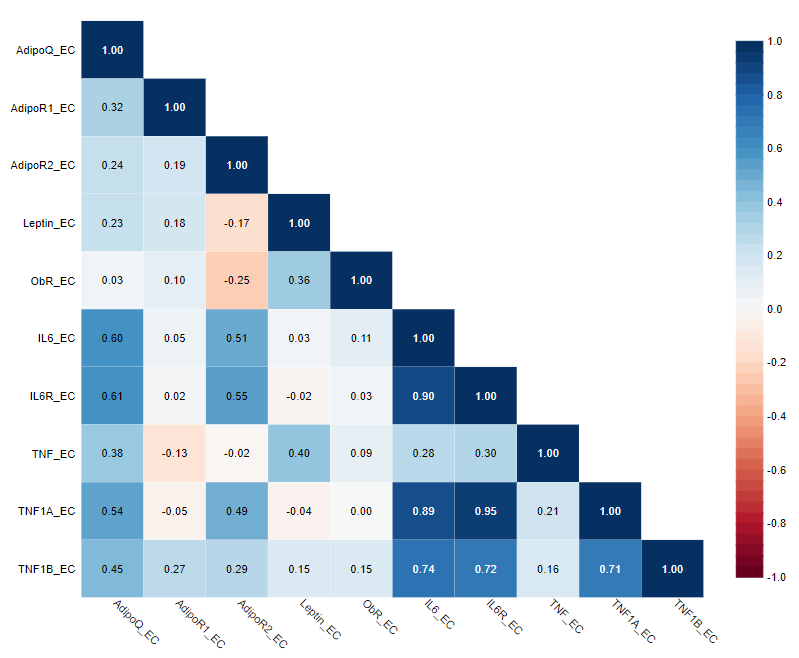


Supplementary Figure 1 Correlation plots between biomarker and their receptor expression in endometrial cancer tissue (EC).

Pearson correlation calculations have been performed and the numbers in the boxes denote Pearson’s r values. Red to blue end of the scale illustrates strong negative (r=-1) to strong positive (r=+1) correlation with white indicating no correlation (r=0).

Also, there was lack of correlations between the expression of adiponectin, leptin and their receptors in adipose tissue and endometrial cancer tissue suggesting that the expression levels of these adipokines in adipose tissue do not directly correspond to their expression in endometrial cancer tissue, implying that contrary to our hypothesis, local parametrial adipose tissue may not be involved in the regulation of these markers in the endometrial cancer tissue. They are perhaps affected by a systemic obese state and some other regulatory mechanisms and microenvironment factors, that influence their expression in the specific context of endometrial cancer.

## Association between biomarker expression and patient demographics and tumour characteristics

(N=9)

(N=22)

(N=7)

(N=32)


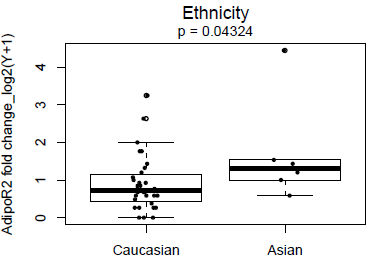

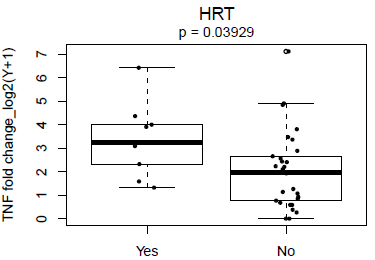


A


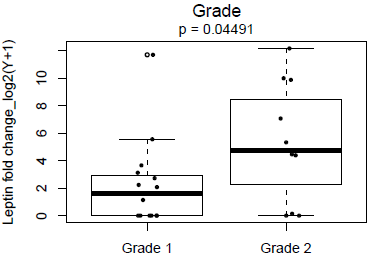

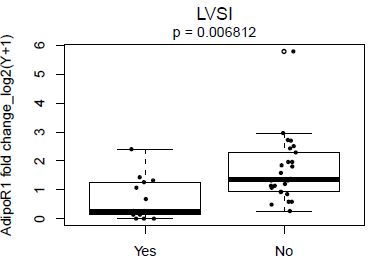


BA

(N=15)

(N=13)

(N=26)

(N=11)

Supplementary Figure 2 Box plots to demonstrate the association between biomarker expression by qPCR and (A) demographic characteristics and (B) cancer characteristics using univariate linear regression. Biomarker expression is expressed as fold change. X axis- demographic factors’/ cancer characteristics’ groups; Y axis- log2 [(fold change) +1]. The 25th and 75th percentiles are represented by the lower and upper boundaries of the rectangles, respectively, while the median is indicated by the horizontal line inside the rectangles. The whiskers extend from the box denoting the minimum and maximum values.

1. **Comparison of expression of the biomarkers between three tissue types**

The average expression of adiponectin, leptin and their receptors are plotted on a heat map (Supplementary Figure 3). Higher expression levels of adiponectin and leptin are observed in both lymph node and adipose tissue, indicating similarities in their expression profiles. Additionally, lymph nodes show higher expression of leptin and lower expression of adiponectin compared to adipose tissue. However, no significant differences are observed in the expression of their receptors.

No significant interrelationship was noted among expression of the markers and their receptors in endometrial cancer tissue (n=39) (Supplementary Figure 4) or, matched adipose tissue and endometrial cancer tissue and matched lymph nodal tissue (n=12) (Supplementary Figure 5).

Supplementary Figure 3 Heat map demonstrating the average expression of adiponectin, leptin and their markers in endometrial cancer tissue, parametrial adipose tissue and lymph nodes. AdipoQ= adiponectin, ADIPOR1/R2=adiponectin receptors, OBR=leptin receptor. Gene expressions are plotted on the Y-axis against the type of tissue on the X-axis. Gene expressions are plotted as mean of fold change over a calibrator sample. The colour scale ranges from black (lowest value) to white(median) to yellow (highest value).


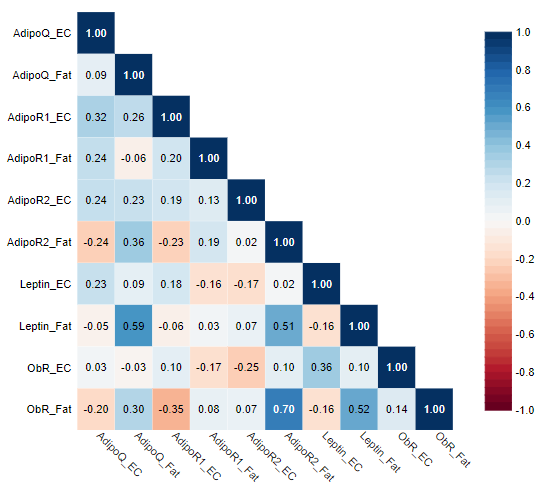


Supplementary Figure 4 Correlation plots comparing expression of biomarkers and their receptors between endometrial cancer tissue (EC) and adipose tissue (Fat) for 39 patients.

Pearson correlation calculations have been used to seek correlation. Red to blue end of the scale illustrates strong negative (Pearson’s r=-1) to strong positive (Pearson’s r=+1) correlation with white indicating no correlation Pearson’s (r=0). The numbers in the boxes denote Pearson’s r values.


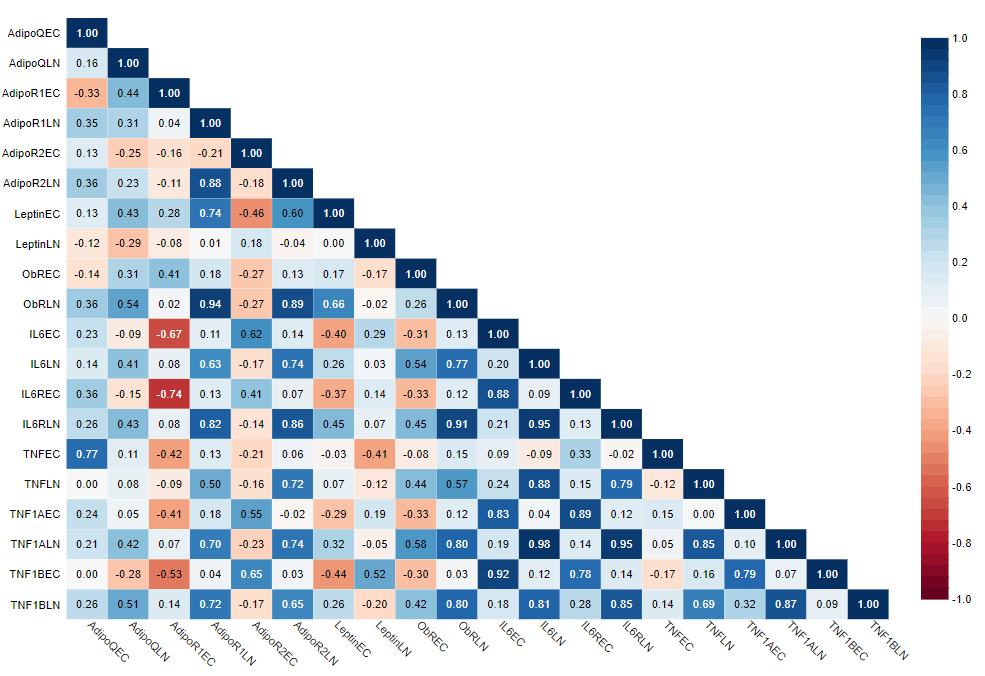
Supplementary Figure 5 Correlation plots comparing expression of biomarkers and their receptors between endometrial cancer tissue (EC) and lymph nodal tissue (LN) for 12 patients.

Pearson correlation calculations have been used and the numbers in the boxes denote Pearson’s r values. Red to blue end of the scale illustrates strong negative (r=-1) to strong positive (r=+1) correlation with white indicating no correlation (r=0).

## Comparison between tissues expression and circulating levels of the biomarkers


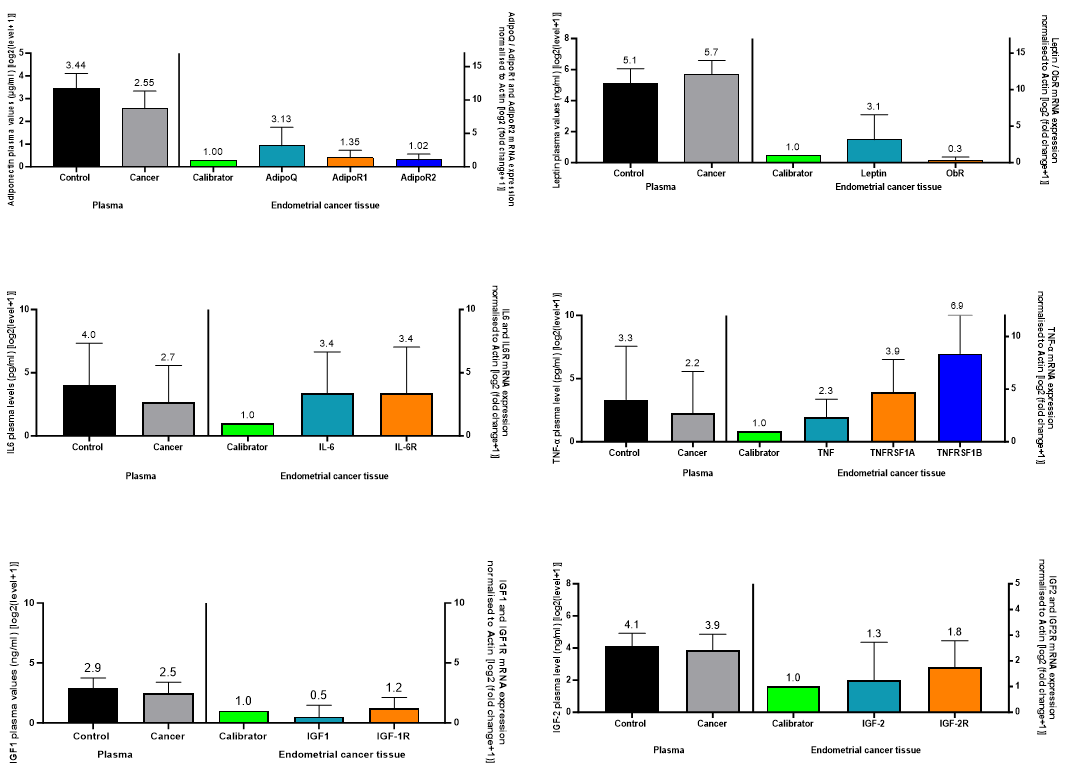


A

B

C


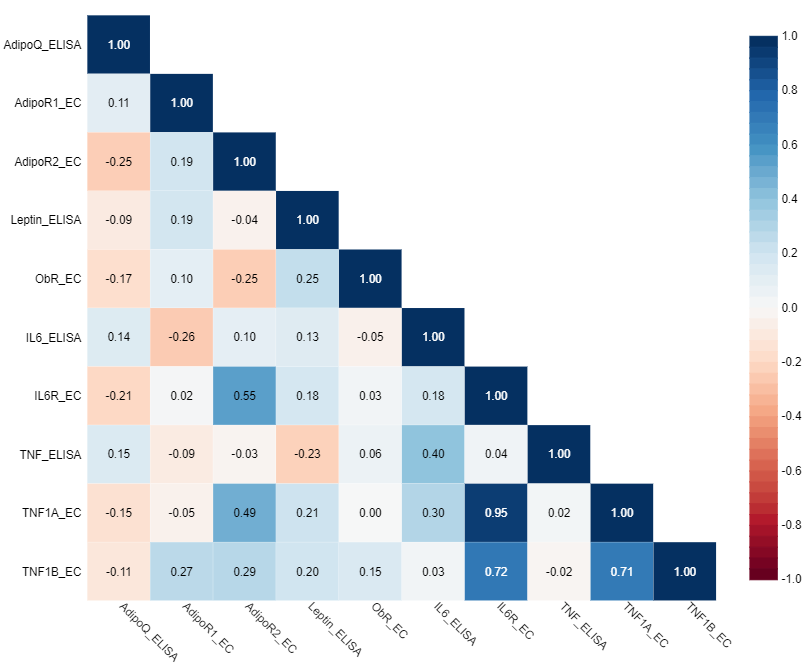

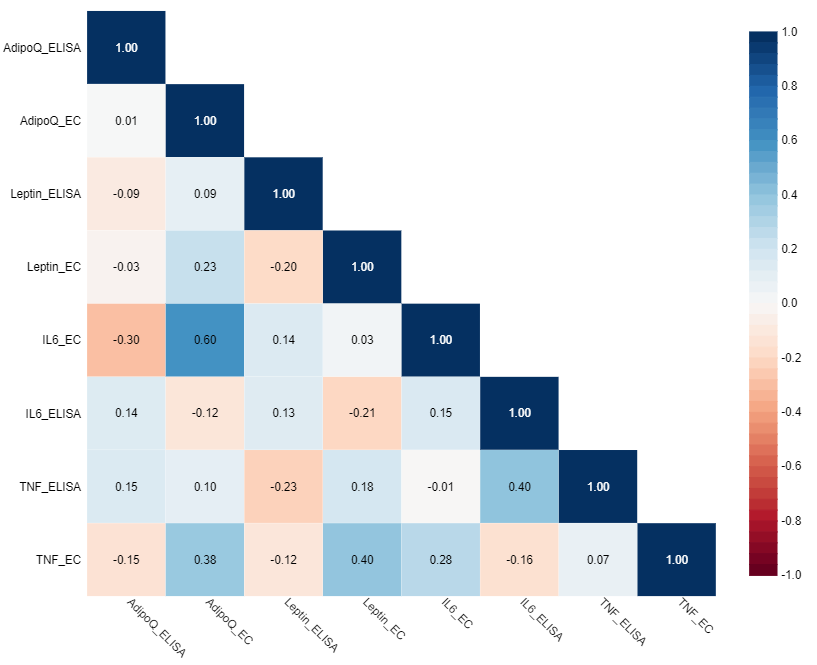


Supplementary Figure 6 (A) Bar graphs demonstrating the comparison of the levels of each biomarker in plasma and endometrial cancer tissue between cancer and control patients.

Two different Y axis have been used for the illustration of plasma and PCR levels. All data has been log transformed [log_2_(Y+1)]. The error bars represent standard error of mean. The receptors of the biomarkers have been measured only in the endomertial cancer tissues and not in plasma. 6 (B and C) Correlation plots between (B) circulating biomarker levels (ELISA) and tissue (PCR) and (C) circulating biomarker levels (ELISA) and their receptors in tissues (PCR).

Each biomarker level in the plasma has been correlated with its tissue expression and its receptor expression in tissues using Pearson correlation calculations and the numbers in the boxes denote Pearson’s r values. Red to blue end of the scale illustrates strong negative (r=-1) to strong positive (r=+1) correlation with white indicating no correlation (r=0).

1. **Validation of protein expression for ADIPOR1 and R2 receptors**

To validate genuine protein expression, positive and negative controls were used for every run of IHC. Human thyroid gland tissue served as the positive control for ADIPOR1, while human placental tissue was employed as the positive control for ADIPOR2. In both cases, the receptors exhibited cytosolic enhancement in their respective positive controls.

Interestingly, one patient had both normal and cancerous endometrial tissue on the same slide. In this case, the normal endometrium displayed a '2+' expression of ADIPOR2, whereas the cancerous portion exhibited a '1+' expression of ADIPOR2, as illustrated in Supplementary Figure 7, demonstrating that AdipoR2 is less expressed in endometrial cancer tissue.

The confirmation of true expression was further substantiated when a tissue section from a benign endometrial sample revealed the expression of the receptors exclusively in the endometrial glands, with no discernible expression in the cervical glands within the same tissue section (Supplementary Figure 8). Furthermore, it is evident that the same benign endometrium expresses more ADIPOR1 (IHC score 2+) than ADIPOR2 (IHC score 1+).


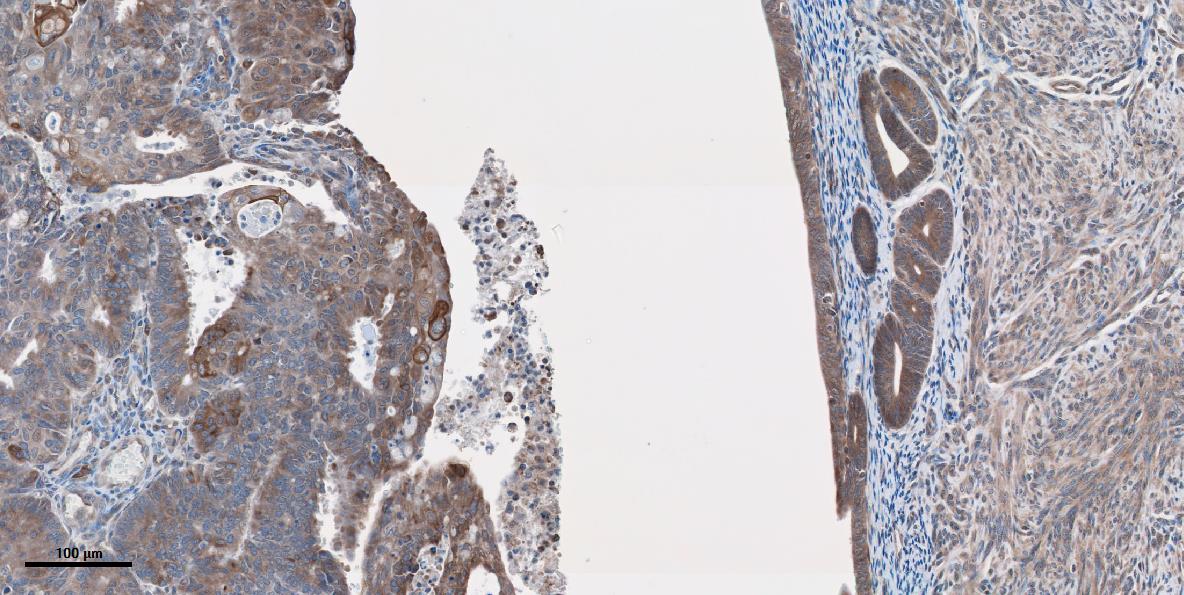


Cancerous endometrium glands


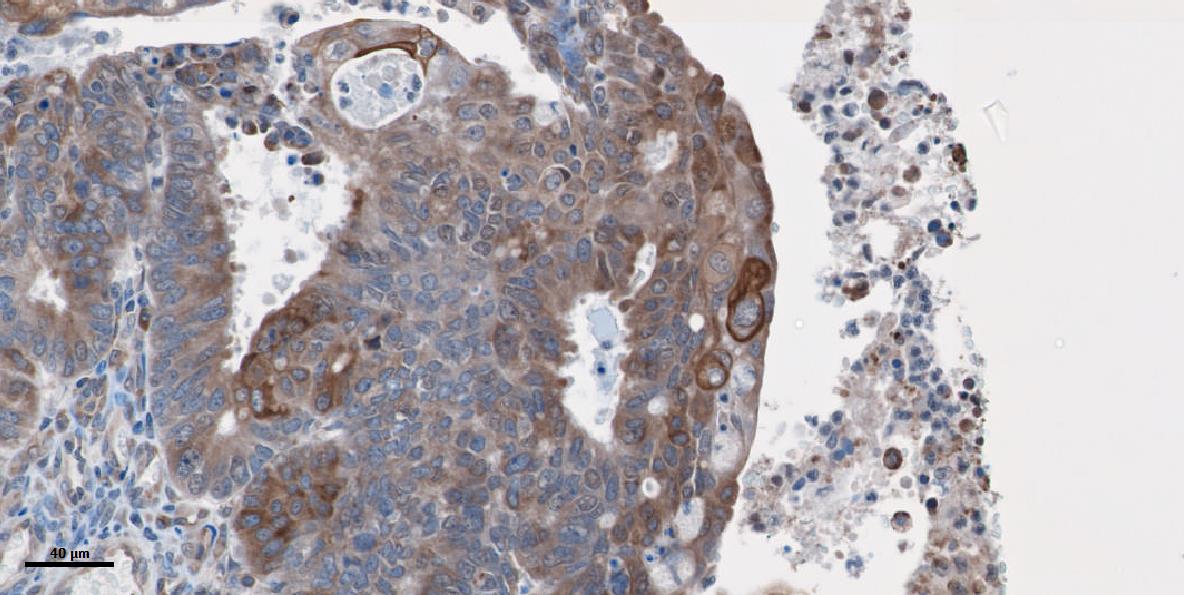


Benign endometrium glands


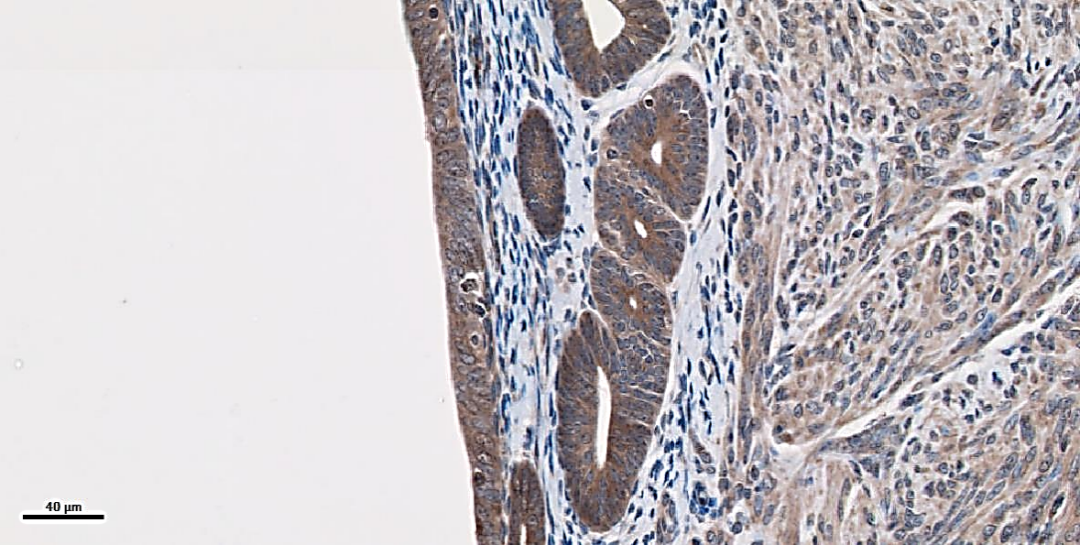


Supplementary Figure 7 Figure illustrating chromogenic 3,3′-Diaminobenzidine (DAB) staining for ADIPOR2 in an endometrial cancer patient.

The cancer tissue section demonstrates the presence of cancerous and benign endometrial tissue in the same slide. ADIPOR2 expression was marked as 2+ in the benign endometrial part and 1+ in the cancerous part. Image taken at 10X magnification, Scale bar = 100 μm.


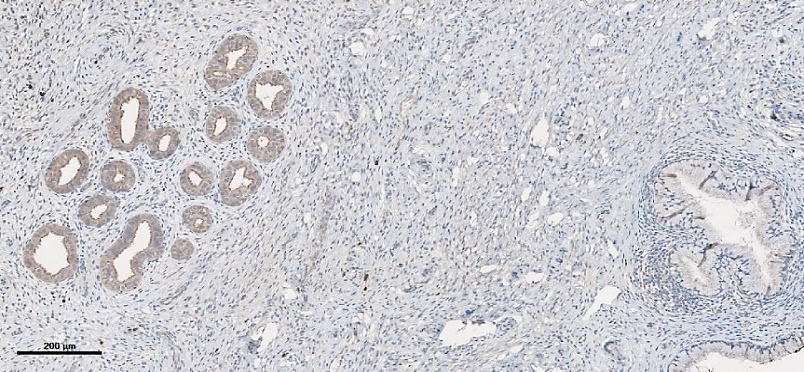

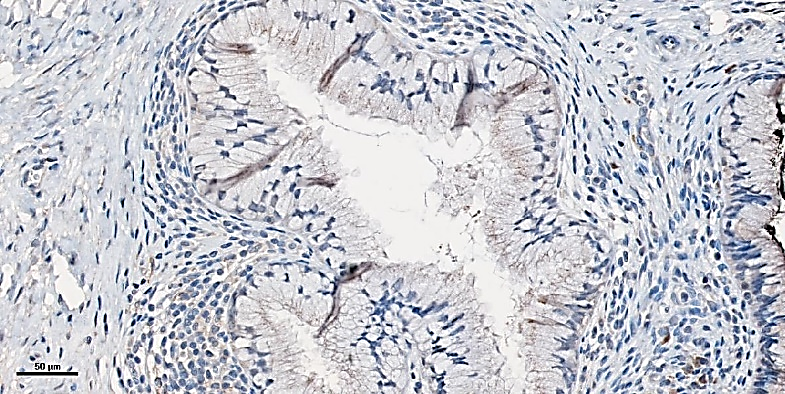


Cervical glands


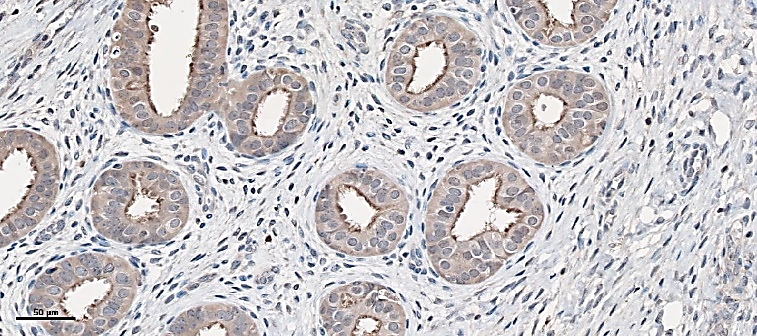


Endometrial glands

ADIPOR2 expression


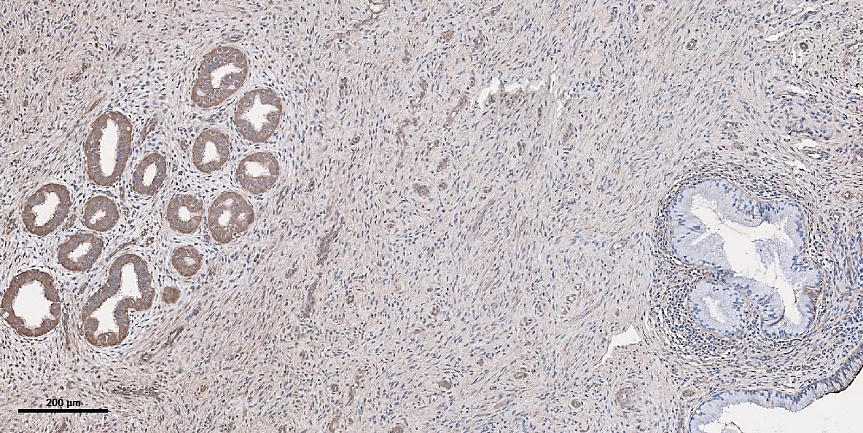


ADIPOR1 expression

Cervical glands


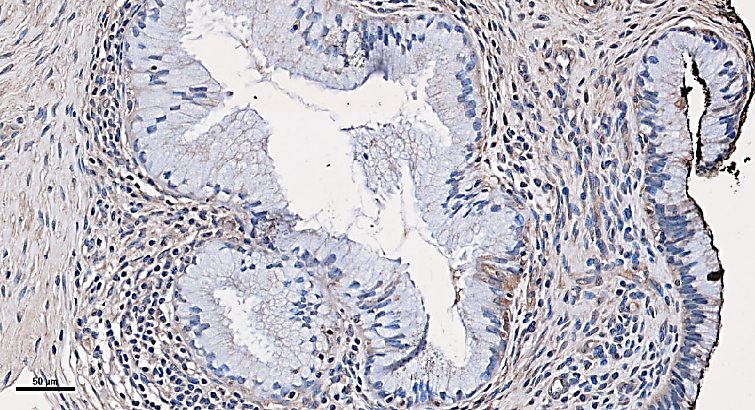


Endometrial glands


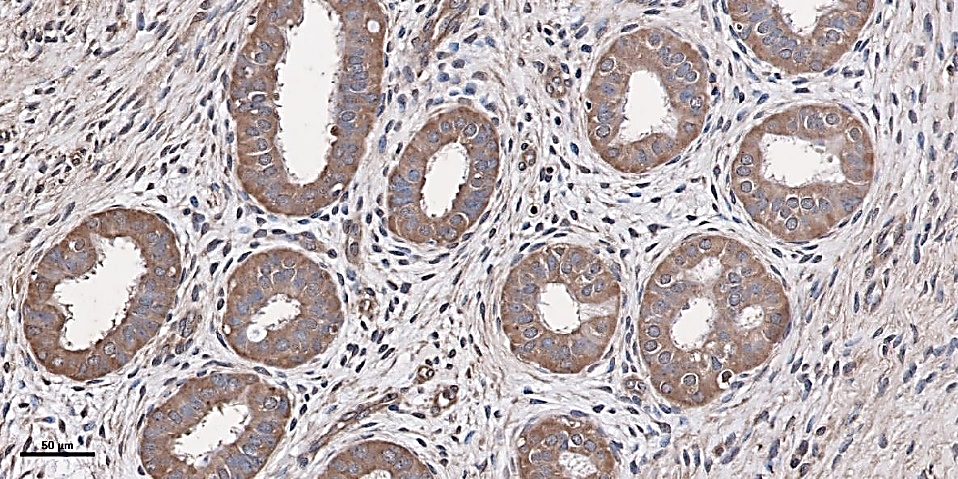


**Supplementary Figure 8 Figure illustrating chromogenic 3,3′-Diaminobenzidine (DAB) staining for ADIPOR1 and ADIPOR2 receptors in benign endometrial sample showing both endometrial and cervical glands in the same tissue section on the slide.**

Image taken at 10X magnification, Scale bar = 200 μm.

1. **Comparison between IHC score and fold change in qPCR**

The IHC scoring and the mRNA expression (fold change) were correlated using Pearson’s correlations. Interestingly, the two levels did not correlate for either marker as the r values for both are 0.1 (Supplementary Figure 9).

**Supplementary Figure 9 Scatter plots demonstrating correlation analysis (Pearson's) between IHC score and qPCR fold change (FC) expression of ADIPOR1 and ADIPOR2.** r= Pearson's r value, p=significant if <0.05.
